# Editorial: Methods in pediatric gastroenterology, hepatology and nutrition

**DOI:** 10.3389/fped.2024.1521649

**Published:** 2024-11-25

**Authors:** Silvia Salvatore, Simona Sestito, Licia Pensabene

**Affiliations:** ^1^ Pediatric Department, University of Insubria, Hospital “F. Del Ponte”, Varese, Italy; ^2^ Pediatric Unit, Department of Medical and Surgical Sciences, University Magna Graecia of Catanzaro, Catanzaro, Italy

**Keywords:** methods, non-invasive technique, diagnostic procedures, ultrasound, elastography, celiac disease diagnosis

**Editorial on the Research Topic**
Methods in pediatric gastroenterology, hepatology and nutrition

Diagnostic methods in gastroenterology and hepatology have evolved greatly in recent years, ranging from accurate and specific blood and genetic tests to sophisticated imaging and histology techniques, each contributing unique benefits to pediatric care.

This special issue of *Frontiers in Pediatrics* aimed to focus on novel findings and new approaches to different pediatric gastrointestinal and liver diseases, highlighting recent diagnostic advances. The four studies included in this issue clearly exemplify three different field of application: celiac disease diagnosis, foreign bodies detection and liver tissue alteration.

The presentation of Celiac disease (CD) may vary from a broad spectrum of gastrointestinal and extraintestinal symptoms, to severe or subclinical cases (1), not significantly changed during the recent years and SARS-CoV-2 pandemic (2). Delayed diagnosis and unrecognized cases still occur despite the availability of a highly sensitive and specific antibody test, such as the tissue transglutaminase antibodies (TTG). Clinicians should be aware that celiac crisis is a rare but critical and acute manifestation of CD in which a prompt diagnosis and treatment are crucial interventions, as it may represent a life-threatening condition (3). In this issue, Mauro et al. reported the case of a 22-month-old child who presented with severe malnutrition, hypoalbuminemia, and dehydration requiring 22 days of hospitalization, intravenous infusion of albumin and steroids and parenteral nutritional support.

Current guidelines on CD in children recommend that the diagnosis should rely on the presence of high-level positivity (>10× upper normal limit) of IgA anti-transglutaminase antibodies (TTG), positive antiendomysial antibodies and/or specific inflammatory and atrophic findings at duodenal biopsy (4). Data on the correlation between TTG levels, genetic markers (HLA-DQ2 and DQ8), and duodenal biopsy findings are limited in children (4, 5). A recent study (Schesquini-Roriz et al.) including 112 young children (mean age 6 ± 4 years) outlined that the higher the TTG levels the more severe intestinal atrophy is present. Avoiding intestinal biopsy, whenever feasible, significantly reduce healthcare costs, discomfort for patients, and eventual delayed diagnosis, malnutrition, growth delays, and other CD complications, particularly in young children. Interestingly, in this cohort the authors found an inverse relation between TTG levels and age of patients at diagnosis and revealed a few cases of CD with negative genetic markers. However, it is worth nothing that HLA testing was performed only with a single diagnostic kit in most subjects and HLA DQ8 was not tested in 27% of the population.

Timely accurate diagnosis is overall an outmost clinical priority in Emergency Departments. Abdominal ultrasound is increasingly used to identify the different causes of abdominal pain or of bowel obstruction and aid in patient management including interventional and surgical planning (6). A real-time point-of-care abdominal ultrasound (abdominal POCUS) has recently shown superiority over an abdominal x-ray to identify a spectrum of critical abdominal pathological conditions in emergency situations in neonates and children (7).

In this issue high-frequency ultrasound has proven effective in detecting the presence and location of foreign bodies in children, with optimal sensitivity in identifying magnets particularly in the stomach (96%) and small bowel (100%) and for gastrointestinal wall entrapment (92%) (Xin et al.). The detailed imaging capabilities of ultrasound enable clinicians to detect gastrointestinal obstructions, offering a non-invasive, reliable tool for quick assessment, reducing the need for additional imaging and exposure to radiation in children.

Likewise, ultrasound elastography is emerging as a new promising technique to assess stiffness of liver and evaluate the degree of fibrosis potentially reducing the need of other diagnostic investigation (i.e., magnetic resonance elastography) and liver biopsy. Hepatic steatosis and fibrosis of various aetiologies are increasingly recognised in the pediatric population (8, 9). Since liver fibrosis may progress to cirrhosis, portal hypertension-related complications, liver failure and hepatocellular carcinoma, accurate and early identification of fibrosis has important prognostic implication (9).

Shear wave elastography (SWE) is a non-invasive technique that utilizes the acoustic radiation force impulse from the ultrasound probe to generate shear wave propagation through the liver parenchyma where the propagation velocity is measured (9). A recent meta-analysis of individual data from 1,134 adult patients with different liver diseases showed a high accuracy (>85%) of 2D-SWE for the detection of fibrosis and cirrhosis (10). SWE has been validated in adult subjects but not yet in young patients. The study included in this issue (Cetiner et al.), reported, for the first time, pediatric normative and percentiles values for attenuation imaging coefficient, SWE and dispersion data that reflect attenuation, elasticity, and viscosity of liver tissue. The authors recruited 129 children (aged 0–18 years) attending outpatient clinics because of minor illnesses excluding fever, liver or cardiac diseases. As important covariates such as age, sex and body mass index have been considered and analysed, these results provide a significant contribution and may aid for the clinical application of SWE and for monitoring pediatric hepatic patients.

The four studies published in this issue highlight the importance of comprehensive and pediatric targeted diagnostic approaches, including serology, genetic tests, and imaging, which together form a holistic strategy to manage complex pediatric cases. In conclusion, the integration of these diagnostic advancements allows for more precise and tailored interventions, underscoring the field evolution toward minimizing invasive procedures and patient discomfort and maximizing clinical outcomes. We hope this special issue will inspire further research and innovation in advanced pediatric diagnostics.
